# Effects of root-colonizing fungi on pioneer *Pinus thunbergii* seedlings in primary successional volcanic mudflow on Kuchinoerabu Island, Japan

**DOI:** 10.1007/s00572-024-01142-y

**Published:** 2024-03-19

**Authors:** Akira Ishikawa, Daisuke Hayasaka, Kazuhide Nara

**Affiliations:** 1 Graduate School of Frontier Sciences, University of Tokyo, 5-1-5 Kashiwanoha, Kashiwa, 277-0882 Chiba Japan; 2 Faculty of Agriculture, Kindai University, 3327-204 Nakamachi, Nara, 631-8505 Nara Japan

**Keywords:** Ectomycorrhizal fungi, Arbuscular mycorrhizal fungi, Dark septate endophyte fungi, Volcanic succession, Japanese black pine

## Abstract

Root-colonizing fungi, such as mycorrhizal fungi and dark septate endophyte fungi, are often found on pioneer plant species during early primary succession. However, little is known about which fungal species are responsible for the establishment of pioneer plants when these symbionts colonize simultaneously. We investigated the root-colonizing fungal communities of *Pinus thunbergii* that established prior to lichens, bryophytes, and short-lived herbaceous plants in a primary successional volcanic mudflow site on Kuchinoerabu Island, Japan. We collected a total of 54 current-year and 1- to 2-year-old seedlings. The colonization of root fungi was evaluated by direct observation of key structures (e.g., mantle, arbuscule, microsclerotia, and hyphae) and molecular analysis. Of the 34 current-year seedlings collected, only 12 individuals were colonized by ectomycorrhizal (ECM) fungi. By contrast, all 1- to 2-year-old seedlings were colonized by ECM fungi. Seedlings colonized by pine-specific ECM fungi, specifically *Rhizopogon roseolus* and *Suillus granulatus*, showed higher nitrogen and phosphorus contents in their needles compared to non-ECM seedlings. Arbuscular mycorrhizal fungi and dark septate endophyte fungi were found in only two and three individuals, respectively. The high density of mycophagous deer on Kuchinoerabu-jima may contribute to the favored dispersal of ECM fungi over other root-colonizing fungi. In conclusion, the seedling establishment of *P*. *thunbergii* at the volcanic mudflow may be largely supported by ECM fungi, with negligible effects of arbuscular mycorrhizal fungi and dark septate endophytes.

**Supplementary Information:**

The online version contains supplementary material available at 10.1007/s00572-024-01142-y.

## Introduction

Terrestrial plants interact with various fungi through their roots (van der Putten et al. 2013; Wardle et al. 2002). Among these interactions, mycorrhizal fungi positively affect host plants by enhancing water and nutrient absorption as well as providing protection from antagonists in exchange for photosynthetic products (Smith and Read 2008). Ectomycorrhizal (ECM) and arbuscular mycorrhizal (AM) fungi are prominent mycorrhizal groups associated with dominant plant species in global ecosystems (Brundrett 2017). Furthermore, dark septate endophyte (DSE) fungi, which form characteristic structures such as melanized mycelia and microsclerotia instead of mycorrhizal structures (Andrade-Linares and Franken 2013; Rodriguez et al. 2009), have been reported from approximately 600 plant species across various ecosystems (Jumpponen 2001; Jumpponen and Trappe 1998). The coexistence of mycorrhizal and DSE fungi in both seedlings and mature trees within forest ecosystems has also been reported in high-throughput sequencing-based studies (Toju et al. 2013; Yamamoto et al. 2014). Therefore, mycorrhizal and DSE fungi are ubiquitous in plant roots, from seedlings to mature trees, in most natural ecosystems.

As mycorrhizal fungi play a crucial role in nutrient absorption by host plants, the composition of their infection source can be a limiting factor for host plant establishment, particularly in primary successional sites where the infection source can be scarce (Allen 1987; Allen et al. 1992). In the initial stage of primary succession, mycorrhizal propagules such as spores and sclerotia must be transported from the surrounding areas. While the dispersal ability of propagules varies among mycorrhizal fungal types and species, they are dispersed by both biotic (e.g., mammals, birds, and invertebrates) and abiotic (e.g., wind and water) vectors (Horton 2017; Paz et al. 2021). Previous studies have reported that mycorrhizal fungal diversity and composition vary with distance from surrounding vegetation (Ashkannejhad and Horton 2006; Cázares et al. 2005; Wu et al. 2007). In addition, mycorrhizal fungi in primary successional sites are often dominated by a few species that can maintain the infectivity of propagules for long periods in harsh environments (Ashkannejhad and Horton 2006; Dickie et al. 2013). The plant species involved in pioneer invasion also influence the composition of mycorrhizal fungi, particularly ECM fungi (Ashkannejhad and Horton 2006; Ishikawa and Nara 2023; Nara et al. 2003). Therefore, the composition of mycorrhizal fungi in primary successional sites would be affected by various factors and vary among sites.

Plants are usually classified as forming a single type of mycorrhiza. However, some plants can form mycorrhizae with both AM and ECM fungi, either simultaneously within the same root system or at different life stages (Molina et al. 1992). Teste et al. (2020) hypothesized that early colonization by AM fungi may confer advantages to seedlings if the carbon cost associated with AM fungi is lower than that of ECM fungi. Studies have reported that typical ECM host tree species can be colonized by AM fungi that form typical structures such as arbuscules and coils in the roots of the seedlings (Dickie et al. 2001; Horton et al. 1998). This dual mycorrhizal status has been observed in situations with limited infection sources of ECM fungi, such as grasslands and initially in secondary succession. Thus, in primary successional sites with limited sources of mycorrhizal fungi, AM fungi may aid the establishment of plant species that are usually associated with ECM fungi. However, most studies conducted in primary successional environments have focused exclusively on a single type of mycorrhizal fungi (Ashkannejhad and Horton 2006; Nara 2006), and none has considered multiple mycorrhizal types.

Although DSE fungi are ubiquitous among various plant species, their ecological role remains unclear as the results of previous inoculation tests vary from positive to negative (Mayerhofer et al. 2013; Newsham 2011). However, DSE fungi are considered to have neutral or positive effects in strongly stressed environments including early primary succession, particularly when mycorrhizal infection sources are limited (Cázares et al. 2005; Fukuchi et al. 2011; Tejesvi et al. 2010). Moreover, DSE fungi promote AM fungal colonization and enhance phosphorus absorption (Della Monica et al. 2015; Gooden et al. 2020; Wagg et al. 2008). DSE fungi can also interact with ECM fungi, although few previous studies have simultaneously assessed colonization rates of DSE and ECM fungi. Horton et al. (1998) observed that DSE fungi rapidly colonized *Pinus muricata* seedlings before mycorrhizal fungi after a forest fire but did not mention the effect on seedling growth. Thus, the interactions between DSE and mycorrhizal fungi and their effects on seedling establishment (e.g., increased biomass and nutrient absorption) during primary succession remain largely unknown.

Studies of primary succession in Japan have been conducted at various volcanic sites (Iida et al. 2021; Kamijo et al. 2002; Ohsawa 1984; Teramoto et al. 2017; Tsuyuzaki et al. 2005). Pioneer herbaceous plant species are often dominated by Polygonaceae, Cyperaceae, and Poaceae throughout the region. By contrast, the pioneer tree species vary among sites and change with altitude and latitude. For example, *Salix* and *Alnus* species are commonly observed in volcanic sites located in northern or high elevational areas, while *Pinus thunbergii* is recognized as a pioneer species after eruptions in southern Japan (Teramoto et al. 2017). *Pinus thunbergii* is also distributed in coastal forests in Japan, Korea, and China, where their ECM fungal communities have been reported in several studies (Matsuda et al. 2009; Obase et al. 2009). However, no previous studies have examined root-colonizing fungi including ECM fungi associated with pioneer *P*. *thunbergii* after volcanic eruptions.

In late May 2015, a large eruption occurred on Kuchinoerabu Island, located in southern Japan. *Pinus thunbergii* is the most dominant pioneer plant on bare areas formed by lahars (i.e., volcanic mudflows caused by rainwater) that occurred after the eruption, establishing in advance of lichens, bryophytes, and short-lived herbaceous plants. These pine seedlings are sporadically and individually established in this volcanic site, providing a unique opportunity to investigate the relationship between seedling establishment and root-colonizing fungi.

In this study, we investigated the effects of root-colonizing fungi, including AM, ECM, and DSE fungi, on the nutrient status of *P*. *thunbergii* seedlings in a volcanic mudflow area on Kuchinoerabu-jima. Both molecular identification and direct microscopic observation approaches were used because direct observation of fungal structures is necessary to accurately quantify fungal infection in plants (Teste et al. 2020).

## Materials and methods

### Study site and field sampling

**Fig. 1:**
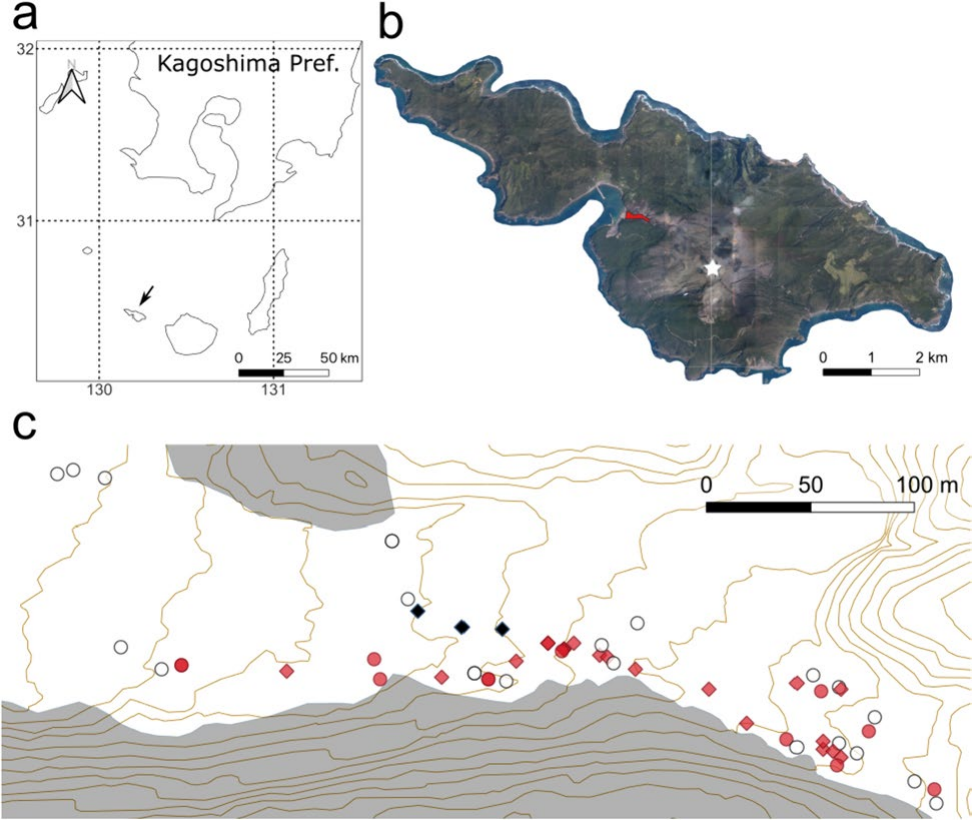
Location of Kuchinoerabu-jima and the study site. **a** Location of Kuchinoerabu-jima (black arrow). **b** Location of the study site (red shading) and crater (white star) on the island. **c** Distribution of *Pinus thunbergii* seedlings at the study site; white circle: current-year non- ECM seedling; red circle: current-year ECM seedlings; red diamond: 1- to 2-year-old ECM seedling; black diamond: mature *P. thunbergii* tree. Shaded areas represent remnant forests

This study was conducted on Kuchinoerabu Island (30°28′ N, 130°12′ E), located ~12 km west of Yakushima Island, Kagoshima Prefecture, southern Japan (Fig. 1). Kuchinoerabu-jima is located in a warm temperate zone with an annual mean temperature of 19.6 °C and annual mean precipitation of 4651.7 mm, according to records from the nearest Yakushima observatory station (from 1991 to 2020; Japan Meteorological Agency: http://www.jma.go.jp). Mount Shindake (626 m above sea level), located on the eastern part of the island, is an active volcano that repeatedly erupts at about 20-year intervals (Tameguri et al. 2016). The last eruption that occurred in 2015 was relatively large, forcing all residents on the island to be evacuated (Tameguri et al. 2016). A pyroclastic flow from this eruption spread west-northwest from the central crater and reached Mukaehama beach. This pyroclastic flow consisted mainly of hot gas, which killed almost all trees; the deposition of ashes was not extensive (Geshi and Itoh 2018). Subsequently, a lahar occurred due to heavy rains after the eruption that deposited ash-mud substrates within a valley toward Mukaehama beach in 2015 (Geshi and Itoh 2018). Lahar deposition with a depth of more than 2 m in the valley formed a bare area ~100 m wide for ~500 m from Mukaehama beach. A pyroclastic flow killed the vegetation on the northern slope of this bare area, but the secondary evergreen forest on the southern slope surrounding the valley remained. Several mature *P*. *thunbergii* trees that formed pinecones also survived in the center of the bare area, located in topographically safe patches.

Pioneer plants in this site include *P*. *thunbergii*, *Toxicodendron succedaneum*, *Miscanthus sinensis*, and *Histiopteris incisa*. These were often observed in the shadows of large rocks where the surface runoff water during heavy rains does not erode the soil substrates. Lichens and bryophytes were scarcely established in this bare area. The secondary evergreen forest on the southern slope of the bare area was mainly composed of *Castanopsis sieboldii*, *Schefflera heptaphylla*, *Ardisia sieboldii*, and *Maesa perlarius* var. *formosana*, and *P*. *thunbergii* was also distributed on the forest edge facing the bare area. In addition, the coastal forest along Mukaehama beach was mainly composed of *P*. *thunbergii*.

In Nov. 2021, we sampled 54 current- to second-year pine seedlings (< 10 cm in height). The sampling points were at least 1 m apart, and their geographical positions were recorded by GPS (Garmin 62 S; Garmin International, Olathe, KS, USA). The collected samples were placed separately in plastic bags and stored at 4 °C until use.

### Measurement of fungal colonization and needle nitrogen and phosphorus

Each seedling was separated into shoot and root parts. Root samples were carefully cleaned of soil and debris with tap water. ECM root tips were classified into morphotypes using a dissecting microscope based on their surface color, shape, texture, and emanating hyphae (Agerer 1991). We collected up to three ECM root tips upon availability for each morphotype per seedling, placed them into separate 2.0 mL tubes containing 50 µL cetyltrimethylammonium bromide (CTAB) buffer, and stored them at −30 °C until use.

After ECM sampling, root samples were divided into halves based on fresh weight and used to measure AM and DSE fungal colonization. Half of the roots were cut into 1–2 cm fragments and autoclaved twice in 10% KCl at 121 °C for 20 min with replacement of the KCl sol. The autoclaved roots were rinsed with tap water and bleached in alkaline hydrogen peroxide (0.5% v/v NH_4_OH and H_2_O_2_; Teste and Laliberté 2018) at 60 °C for 45 min. After rinsing the breached roots with tap water, they were immersed in 2% HCl for 30 min. Finally, the acidified roots were stained using a 0.05% Trypan blue solution at 90 °C for 15 min. AM and DSE fungal colonization were identified by the presence/absence of AM structures (e.g., hyphae, arbuscule, and vesicle) and endophyte structures (e.g., hyphae and microsclerotia) using a light microscope (Eclipse E600; Nikon, Tokyo, Japan).

The other half of the roots were oven-dried at 70 °C for 48 h with the shoot sample and weighed. Dried root samples were stored at −30 °C until molecular extraction. Dried needle samples were digested with sulfuric acid and hydrogen peroxide (Lindner and Harley 1942). Nitrogen and phosphorus concentrations were determined by the indophenol blue method and the molybdenum blue method, respectively (Rodriguez et al. 1994; Scheiner 1976). The nitrogen and phosphorus contents of the needles were calculated from the nitrogen or phosphorus concentration per sample and the dry weight of the needles, excluding the stem.

### Molecular analyses and fungal identification

We extracted DNA from each ECM root tip using the CTAB method (Nara et al. 2003). DNA was also extracted from 20 mg of fine roots collected from each of the dried root samples using a DNeasy Plant Mini Kit (Qiagen, Hilden, Germany) following the manufacturer’s protocol. The internal transcribed spacer (ITS) region of rDNA from the ECM root tip was amplified by polymerase chain reaction (PCR) using the primers ITSOF-T (forward) and LB-W (reverse) (Tedersoo et al. 2008). For unamplified ECM samples, another reverse primer (ITS4) was used (Tedersoo et al. 2008; White et al. 1990).

For the dried root samples from which DSE fungal structures were confirmed, primers ITSOF-T and ITS4 were used to amplify DSE fungi. Similarly, for dried root samples with confirmed AM structures, the small subunit (SSU) rDNA of AM fungi was amplified using the primers AML1 and AML2 (Lee et al. 2008). PCR was performed using an Emerald Amp PCR Master Mix Kit (Takara Bio, Shiga, Japan) under the following conditions: 30 cycles of 98 °C for 10 s, 56 °C for 30 s, and 72 °C for 60 s. To detect DSE and AM fungi in roots where no fungal structures were observed under the microscope, seven dried root samples were randomly selected from microscopically uncolonized seedlings and subjected to the same PCR amplification for DSE and AM. DNA amplification was confirmed by 2% agarose gel electrophoresis.

Amplified products of ECM and AM fungi were purified using ExoSAP-IT (Applied Biosystems, Foster City, CA, USA) and subjected to direct sequencing on a 3730xl DNA Analyzer (Applied Biosystems). Sequencing reactions were performed using a BigDye Terminator v3.1 Cycle Sequencing Kit (Applied Biosystems) with primers ITS1 or ITS4 (ITS regions), or AML1 or AML2 (SSU regions). Because direct sequencing was unsuccessful for DSE products, they were cloned using the Mighty TA-cloning Kit (Takara Bio) following the manufacturer’s protocol. For each DSE product, 12 positive colonies were selected and subjected to PCR with primers ITSOF-T and ITS4 under the same conditions as the initial amplification. Products of colony PCR were purified and sequenced in the same way as ECM and AM fungi.

The sequences of the ITS or SSU regions were assembled after manual trimming and correction using Sequence Scanner Software 2 (ver. 2.0; Applied Biosystems) and ATGC ver. 7 (Genetyx, Tokyo, Japan). High-quality sequences ≥ 250 bp were clustered into operational taxonomic units (OTUs) based on ≥ 97% identity threshold in the VSEARCH program (Rognes et al. 2016). OTU clustering with ≥ 98.5% identity threshold was also performed, because the most frequently used 97% threshold could be too conservative for species-level classification (Nilsson et al. 2019). The taxonomic identities of the clustered consensus sequences, as well as the unclustered sequences with ≥ 350 bp, were determined by BLAST searches against the International Nucleotide Sequence Database Collaboration (INSDC) and UNITE databases (Kõljalg et al. 2005). For DSE fungal identification, OTUs with DSE fungi in the UNITE Species Hypotheses database (at the 1.5% distance threshold; ver. 9 release 2022.10.17) closest to the query sequence were considered DSE fungi, while non-DSE fungi were excluded in this study. The identified ITS and SSU sequences have been deposited in the DNA Data Bank of Japan under accession numbers LC786761–LC786774.

### Data analyses

The sample size-based rarefaction and extrapolation curve as well as the Chao2 richness estimator for ECM fungi were calculated for both current-year and 1- to 2-year-old seedlings using Estimate S ver. 9.1 (Colwell et al. 2012) with 1000 randomizations. All analyses described below were performed with R ver. 4.2.2 (R Core Team 2022). The frequency of ECM fungal species between current-year and 1- to 2-year-old seedlings was compared using Fisher’s exact test. The effects of seedling age and ECM, AM, and DSE fungal colonization on pine seedling dry weight, shoot/root ratio, and needle nitrogen or phosphorus contents were analyzed using a generalized linear model (GLM) with gamma error distributions and a log link function. The full model (response variables ~ Age + ECM + AM + DSE) was simplified to select the best model based on Akaike’s information criterion using the “StepAIC” function from the MASS package (Venables and Ripley 2002). In addition, seedling growth conditions and nutrient contents were compared among the major ECM fungal lineages (i.e., *Amphinema*, suilloid [*Rhizopogon* and *Suillus*], and uncolonized) by Tukey’s *post hoc* test with the “glht” function in the multcomp package for current-year and 1- to 2-year-old seedlings, respectively (Hothorn et al. 2008), while pine seedlings that were colonized by multiple EM fungal OTUs were excluded from this analysis because of the difficulty in isolating the effect of each EM fungus. We also analyzed the correlation between distance from remnant vegetation and colonization by root-colonizing fungi or seedling age using a GLM with binomial error distribution and logit link function.

## Results

We collected 34 current-year seedlings and 20 1- to 2-year-old seedlings (Table 1). Among the current-year seedlings, 12 individuals were colonized by ECM fungi, while the remaining 22 were not. By contrast, ECM fungi colonized all 1- to 2-year-old seedlings. The ECM colonization tended to be located in close proximity to the remaining vegetation, specifically, within a 35 m distance, although no significant correlation was observed between ECM and non-ECM seedling distribution by distance (*P* = 0.48). Similarly, no significant correlation was found between seedling age and proximity to the remaining vegetation (*P* = 0.94).

After morphotyping, we collected 37 and 62 ECM root tips from current-year and 1- to 2-year-old seedlings, respectively. Subsequent sequencing of the 99 ECM root tips yielded 85 high-quality sequences (≥ 250 bp), all of which belonged to ECM fungi and were grouped into nine OTUs at both the 97% and 98.5% identity thresholds (Table 1). *Suillus granulatus* was the most abundant species, detected in eight individuals, followed by *Rhizopogon roseolus* and *Amphinema* sp.1, each found in seven individuals (Table 1).

**Fig. 2:**
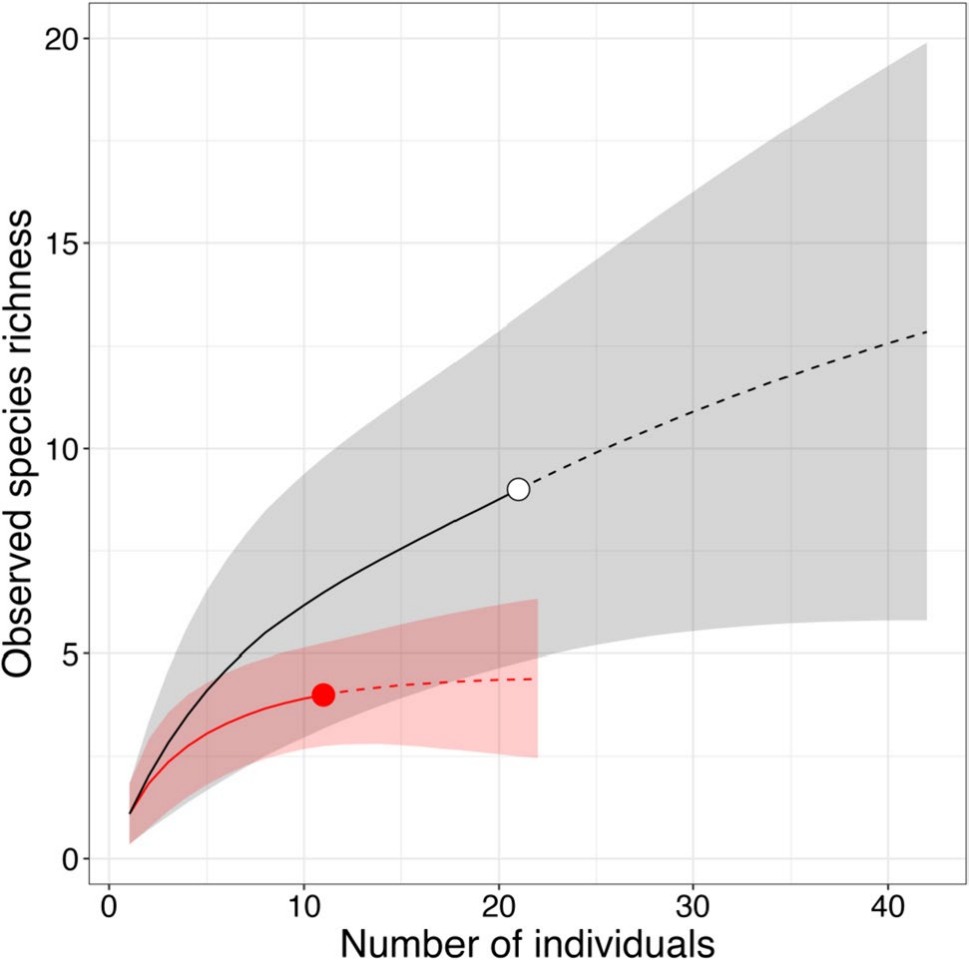
Sample-based rarefaction curves for ECM fungi on current-year (filled red) and 1- to 2-year-old (open black) seedlings of *Pinus thunbergii* in a primary successional volcanic mudflow site 6 years after the 2015 eruption on Kuchinoerabu-jima. Dotted lines indicate extrapolations. Shaded areas represent 95% confidence intervals

A total of four ECM fungal species were identified from current-year seedlings, whereas nine were identified from 1- to 2-year-old seedlings. Rarefaction and extrapolation curves for observed ECM fungal species in this study reached a plateau for current-year seedlings but not for the others (Fig. 2). The Chao2 richness estimators were 4 and 18, respectively. Fisher’s exact tests indicated that the frequency of ECM fungal species significantly differed between the groups (Table 1). *Amphinema* sp.1 was most frequently detected in current-year seedlings, followed by *R*. *roseolus*. By contrast, *S*. *granulatus* was the most frequently detected species in 1- to 2-year-old seedlings, followed by *R*. *roseolus* and *Amphinema* sp. 2.

**Table 1:** Species of ECM, AM, and DSE fungi and their frequencies on naturally establishing *Pinus thunbergii* seedlings in a primary successional volcanic mudflow site 6 years after the 2015 eruption on Kuchinoerabu-jima

Fungal OTUs	Accession No.	Best blast match	DOI of	Identity	Query	Number of *P. thunbergii* seedlings		
		Accession No.	Species hypothesis		length	Current year	1 to 2 years	Total
ECM								
*Amphinema* sp.1	LC786761	AB873189	SH0983710.09FU	99.6	550	6	1	7
*Amphinema* sp.2	LC786762	LC033907	SH1286586.09FU	97.6	653		4	4
*Amphinema* sp.3	LC786763	MN549469	SH1286586.09FU	99.8	438		3	3
*Amphinema* sp.4	LC786764	AB587731	SH1235689.09FU	99.6	479	1	1	2
*Amphinema* sp.5	LC786765	LC623206	SH0929250.09FU	100	457		1	1
*Cenococcum* sp.	LC786768	LC364260	SH0990779.09FU	99.6	463		1	1
*Pisolithus orientalis*	LC786769	UDB009034	SH1251801.09FU	99	404		1	1
*Rhizopogon roseolus*	LC786766	LC198723	SH1210944.09FU	99.7	644	3	4	7
*Suillus granulatus*	LC786767	MN294845	SH1212955.09FU	99.5	651	2	6	8
AM								
*Rhizophagus* sp.	LC786774	FJ009604	SH0988837.09FU	96.9	634	1	1	2
DSE								
Pleosporales sp.	LC786773	MH038071	SH0904803.09FU	97.7	517	1		
Helotiales sp.	LC786772	KP866124	SH0966673.09FU	95.6	594	1		
*Phialocephala fortinii*	LC786771	HM595526	SH0936085.09FU	96.8	502	1	1	2
*Hyaloscypha* sp.	LC786770	JX857212	SH1277500.09FU	97.6	584	1	1	2

**Fig. 3:**
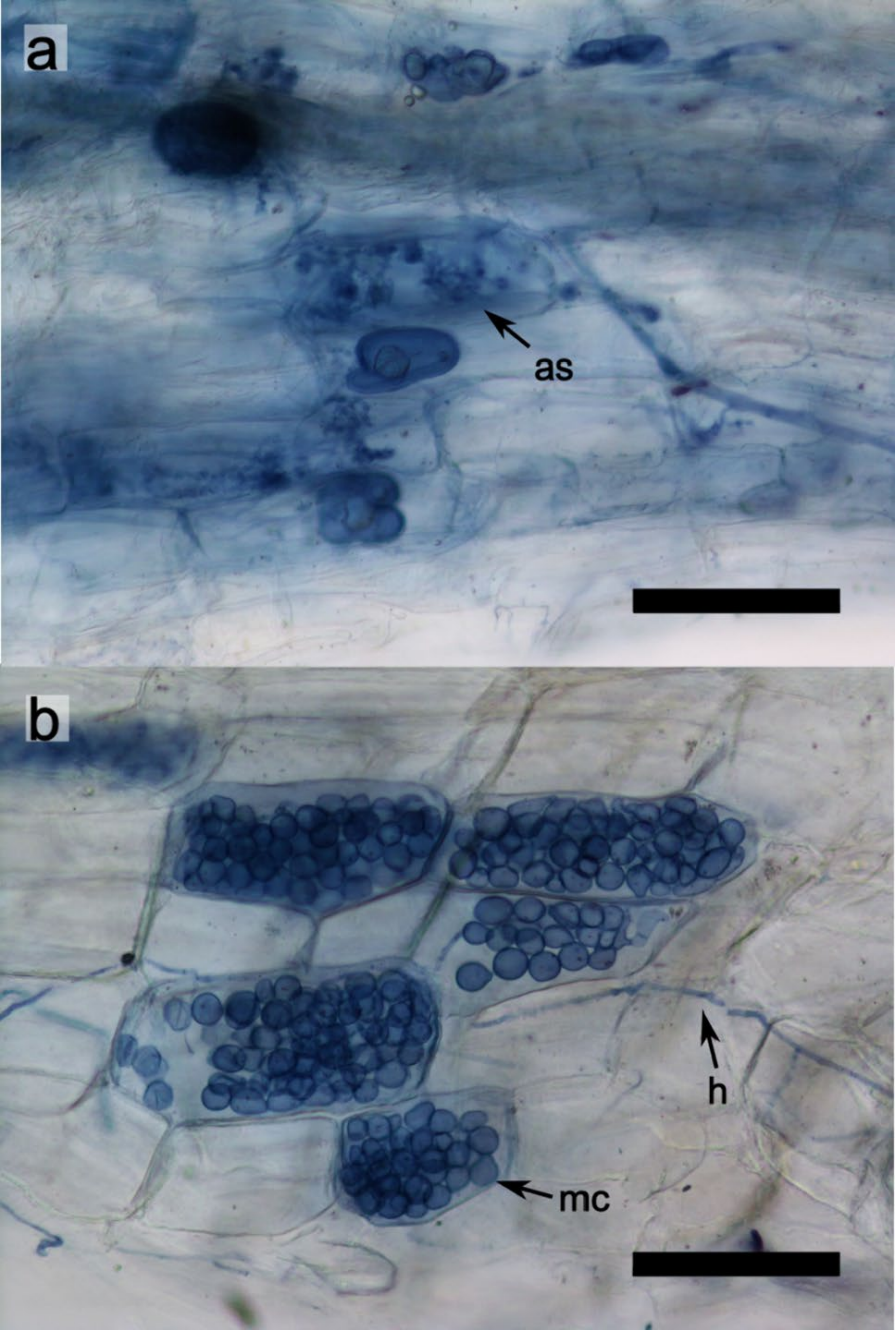
Structures of root-colonizing fungi on *Pinus thunbergii* seedlings in a primary successional volcanic mudflow site 6 years after the 2015 eruption on Kuchinoerabu-jima. **a** AM fungal structures (as). **b** DSE fungal hypha (h) and microsclerotium (mc). Scale bar: 100 μm

AM fungal structures were observed in 2 of 54 pine seedlings, one from a current-year seedling and the other from a 1- to 2-year-old seedling (Fig. 3). Both seedlings were simultaneously colonized by ECM fungi in other root tips. All AM fungal sequences obtained from these seedlings were clustered into a single OTU, *Rhizophagus* sp., at both the 97% and 98.5% identity thresholds. No valid AM sequences were obtained from the seven randomly selected root samples with no observable fungal colonization.

DSE fungal colonization was confirmed in three individuals, one from a current-year seedling and two from the other seedlings, with distinctive microsclerotium and septate hyphae observed under the microscope. While the current-year DSE seedling was not accompanied by any ECM fungus, the two other seedlings were simultaneously colonized by ECM fungi. After sequencing, a total of four DSE fungi were identified from microscopically confirmed roots at both the 97% and 98.5% identity thresholds. One to three DSE fungal OTUs were identified for each colonized seedling. Although some sequences of non-DSE fungal species, including yeasts and common soil fungi, were obtained from the randomly selected root samples lacking fungal structures, they were excluded from further analyses.

**Fig. 4:**
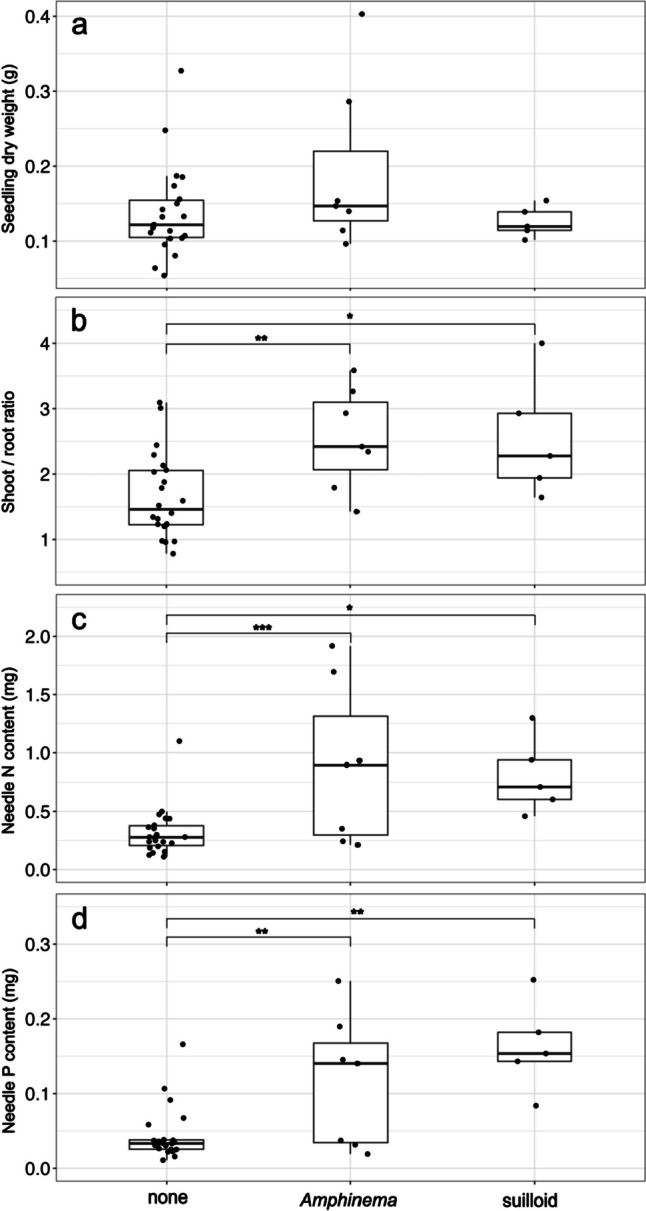
Effects of ectomycorrhizal colonization on the growth and nutrient status of current-year *Pinus thunbergii* seedlings in a primary successional volcanic mudflow site 6 years after the 2015 eruption on Kuchinoerabu-jima (Tukey’s post hoc tests: *P < 0.05, **P < 0.01, ***P < 0.001). **a** Seedling dry weight; **b** shoot/root ratio; **c** needle nitrogen content; **d** needle phosphorus content; none: no ECM colonization. The *Amphinema* and suilloid lineages include two *Amphinema* species and *Rhizopogon roseolus*/*Suillus granulatus*, respectively

Seedling age significantly influenced seedling dry weight and needle nitrogen and phosphorus but not the shoot/root ratio (Table 2). In GLM analyses, ECM colonization significantly increased shoot/root ratios and needle nitrogen and phosphorus contents. The effect of DSE fungal colonization on needle nitrogen and phosphorus contents was marginally significant but the contribution was less than that explained by ECM colonization. In pairwise comparisons, both *Amphinema* species and suilloid species (*S*. *granulatus* and *R*. *roseolus*) increased shoot/root ratios and needle nitrogen and phosphorus contents more than non-ECM fungi in current-year seedlings (Fig. 4). In 1- to 2-year-old seedlings, growth and nutrient status did not significantly differ between *Amphinema* and suilloid species (Fig. S1).

**Table 2:** The effects of seedling age and ECM, AM, and DSE fungal colonization on *Pinus thunbergii* seedlings in a primary successional volcanic mudflow site 6 years after the 2015 eruption on Kuchinoerabu-jima

Response variable	Explanatory variable	Estimate	Standard deviation	*t* value	*P *value
Seedling dry weight	Age	1.037	0.133	7.791	< 0.001
Seedling shoot /root ratios	ECM	0.478	0.120	4.002	< 0.001
Needle nitrogen contents	Age	0.776	0.216	3.549	0.001
	ECM	1.116	0.255	4.532	< 0.001
	DSE	0.774	0.453	1.944	0.094
Needle phosphorus contents	Age	0.970	0.220	4.400	0.001
	ECM	1.287	0.260	5.046	< 0.001
	DSE	0.928	0.463	4.942	0.050

Only explanatory parameters of selected models based on Akaike’s information criterion are shown.

## Discussion

Nearly 65% of current-year seedlings collected at the volcanic mudflow site on Kuchinoerabu-jima were not colonized by mycorrhizal or DSE fungi. This indicates that soil propagule banks of root-colonizing fungi have yet to develop ubiquitously in this primary successional site. While 6 years had passed since the last eruption, this duration may not be sufficient to develop soil propagule banks. Unstable volcanic mudflow substrates that easily move by rainwater would make propagule accumulation in the soil more difficult. In contrast to the current-year seedlings, all of the 1- to 2-year-old seedlings were colonized by ECM fungi. This suggests that, of the current-year seedlings, only those colonized by ECM fungi would survive the winter, whereas most non-ECM seedlings would die out due to nutrient deficiency. Another possibility is that enough ECM fungal spores are dispersed every year from the neighboring forests that non-colonized current-year seedlings would develop ECM symbiosis within a year. A previous study in a heathland also reported that invading pine seedlings are able to remain non-mycorrhizal for a few years and await compatible ECM spores to colonize before starting growth (Collier and Bidartondo 2009).

ECM fungi observed in this volcanic site were dominated by *Rhizopogon*, *Suillus*, and *Amphinema* (Table 1). These ECM fungal lineages have been reported from pine seedlings at invasion forefronts in non-native regions (Policelli et al. 2019) as well as in a recently stabilized sand dune (Ashkannejhad and Horton 2006). Thus, propagules of these fungi should have good dispersal abilities. Indeed, *Suillus* spores can be dispersed more than 1 km by wind (Hynson et al. 2013; Peay et al. 2012), and probably further in the prevailing strong winds on this oceanic island. Furthermore, viable spores of suilloid and *Amphinema* spores can be dispersed up to hundreds of meters by mycophagous deer, as evidenced by successful ECM formation on pine seedlings with fecal matter inoculation (Ashkannejhad and Horton 2006; Nuñez et al. 2013). Kuchinoerabu-jima has a substantial population of *Cervus nippon yakushimae* (Ishida 2019), known to forage on litter and fungi (Agetsuma et al. 2011). Thus, they may contribute to the dispersal of ECM fungal spores from the surrounding remnant forest into the volcanic mudflow site, particularly hypogeous taxa such as *Rhizopogon*.

Compared to ECM colonization, there were much fewer cases of AM and DSE colonization, observed on 2 and 3 of 54 pine seedlings, respectively. This indicates that propagules of these fungi are less available to pine seedlings than ECM fungi at this volcanic site. In a secondary successional site after a forest fire, AM and DSE fungal colonization on regenerating pine seedlings was more frequent (Horton et al. 1998). Thus, while propagules of these fungi in soil could survive the fire, such propagules are not available in volcanic substrates. In the case of AM fungi, spore sizes are far larger than ECM fungi, which makes dispersal with wind to primary successional sites more difficult (Allen et al. 2005; Cázares et al. 2005). Furthermore, Wagg et al. (2008) suggested that AM fungi on Pinaceae, typically an ECM host plant, represent secondary colonization from hyphae of neighboring AM plants. Because pine seedlings in this volcanic mudflow site establish solitarily without neighboring AM plants, the absence of such secondary colonization may also partly account for the lower AM colonization. While little is known about the dispersal of DSE fungi, studies have suggested that fragmented mycelia and microsclerotia can be dispersed and serve as infection sources (Jumpponen and Trappe 1998; Yu et al. 2001). Furthermore, some studies have identified DSE fungal taxa within airborne fungal communities (Kauserud et al. 2005; Kivlin et al. 2014), probably in adhesion to soil particles (Jumpponen et al. 2017). In any case, such dispersal mechanisms should be infrequent at this primary successional site.

ECM colonization significantly increased nitrogen and phosphorus levels in needles of current-year seedlings; ECM seedlings had 2.6- to 2.7-fold higher nitrogen and 2.6- to 3.6-fold higher phosphorus levels than non-ECM seedlings. Suilloid and *Amphinema* species, both dominant in this volcanic site, develop extensive rhizomorphs (Agerer 2001), enabling efficient exploration of soil nutrients. Moreover, suilloid hyphae produce organic acids, solubilizing mineral nutrients from the rock surface (Casarin et al. 2004; Courty et al. 2010; van Schöll et al. 2008). Increased shoot/root ratio by ECM colonization would also indicate improved nutrient absorbing abilities with the extensive ECM mycelial systems, as seedlings can invest more carbon into shoots (Colpaert et al. 1996; Wallander 2000). Extensive rhizomorphs formed by these fungi also promote water absorption and transport (Duddridge et al. 1980; Parke et al. 1983), potentially contributing to higher survival rates under the frequent drought stress in volcanic substrates. Considering the dominance of these ECM fungi in the naturally established seedlings and the enhanced nutrient status by their colonization, they should play critical roles in pine seedling establishment in this primary successional site. While ECM fungal species on pioneer trees vary among volcanic sites (e.g., generalists such as *Laccaria* and *Inocybe* on *Salix* on Mts. Fuji and Usu [Nara et al. 2003; Obase et al. 2007] and specialists such as *Alpova* on *Alnus* seedlings on Izu-Oshima [Ishikawa and Nara 2023]), positive effects of ECM fungi on the establishment of pioneer seedlings are consistently supported (Dickie et al. 2013).

Compared to ECM fungi, colonization by AM or DSE fungi was far less frequent, even in 1- to 2-year-old seedlings. Moreover, the effects of AM and DSE fungi on seedling nutrient status remained nonsignificant and marginally significant, respectively. These findings indicate that AM and DSE fungi have negligible roles in pine seedling establishment at this volcanic site. In experimental conditions, AM fungal colonization has been shown to increase the foliar phosphorus concentration of Douglas fir seedlings (Smith et al. 1998); however, no previous studies have reported positive effects of AM colonization on typically ECM-host species under natural conditions. While direct evidence of DSE fungal functions in seedling establishment processes in the field is also unavailable, mineralization and solubilization of organic compounds by extracellular enzymes produced by DSE fungi could potentially benefit seedlings (Knapp and Kovács 2016; Mandyan and Jumpponen 2005; Newsham 2011). As host responses vary with DSE/mycorrhizal colonization ratios (Mandyan and Jumpponen 2005; Reininger and Sieber 2012; Xie et al. 2021), further colonization by DSE fungi with the development of soil propagule banks may potentially contribute to seedling establishment in the future.

In conclusion, we confirmed the colonization of AM and DSE fungi on naturally established pine seedlings in a primary successional site after the volcanic eruption, although their contribution to seedling nutrient status and growth remained insignificant with the extremely low frequencies of the colonized seedlings. In contrast, all 1- to 2-year-old seedlings and about 1/3 of the current-year seedlings were colonized by ECM fungi, dominated by *Amphinema* spp. and suilloid fungi with the extensive mycelial exploration type. Leaf nitrogen and phosphorus contents of the current-year seedlings colonized by these ECM fungi were far more than double that of the uncolonized seedlings of the same age. Our results indicate that these ECM fungi, not AM and DSE fungi, play the primary role as symbiotic microbes in the seedling establishment of the pioneer pine in this volcanic site.

### Supplementary Information

Below is the link to the electronic supplementary material.

Supplementary file1 (PDF 242 KB)

## Data Availability

No datasets were generated or analysed during the current study.
