# Supplementary material for: Effects of root-colonizing fungi on pioneer Pinus thunbergii seedlings in primary successional volcanic mudflow on Kuchinoerabu Island, Japan
Source: Mycorrhiza. 2024 Mar 19;34(1-2):57–67. doi: 10.1007/s00572-024-01142-y (PMC10998786; doi:10.1007/s00572-024-01142-y)
Supplement: Supplementary file 1 — Supplementary file1 (PDF 242 KB) [file 572_2024_1142_MOESM1_ESM.pdf]

Article title: Effects of root-colonizing fungi on pioneer *Pinus thunbergii* seedlings in primary successional volcanic mudflow on Kuchinoerabu Island, Japan

Journal name: Mycorrhiza

Author names: Akira Ishikawa[1], Daisuke Hayasaka[2] and Kazuhide Nara[1]

Affiliation: [1]Graduate School of Frontier Sciences, University of Tokyo, 5-1-5, Kashiwanoha, Kashiwa, Chiba 277-0882, Japan

[2]Faculty of Agriculture, Kindai University, 3327-204, Nakamachi, Nara, Nara 631-8505, Japan

e-mail: ishikawaa95@gmail.com

Supplementary materials

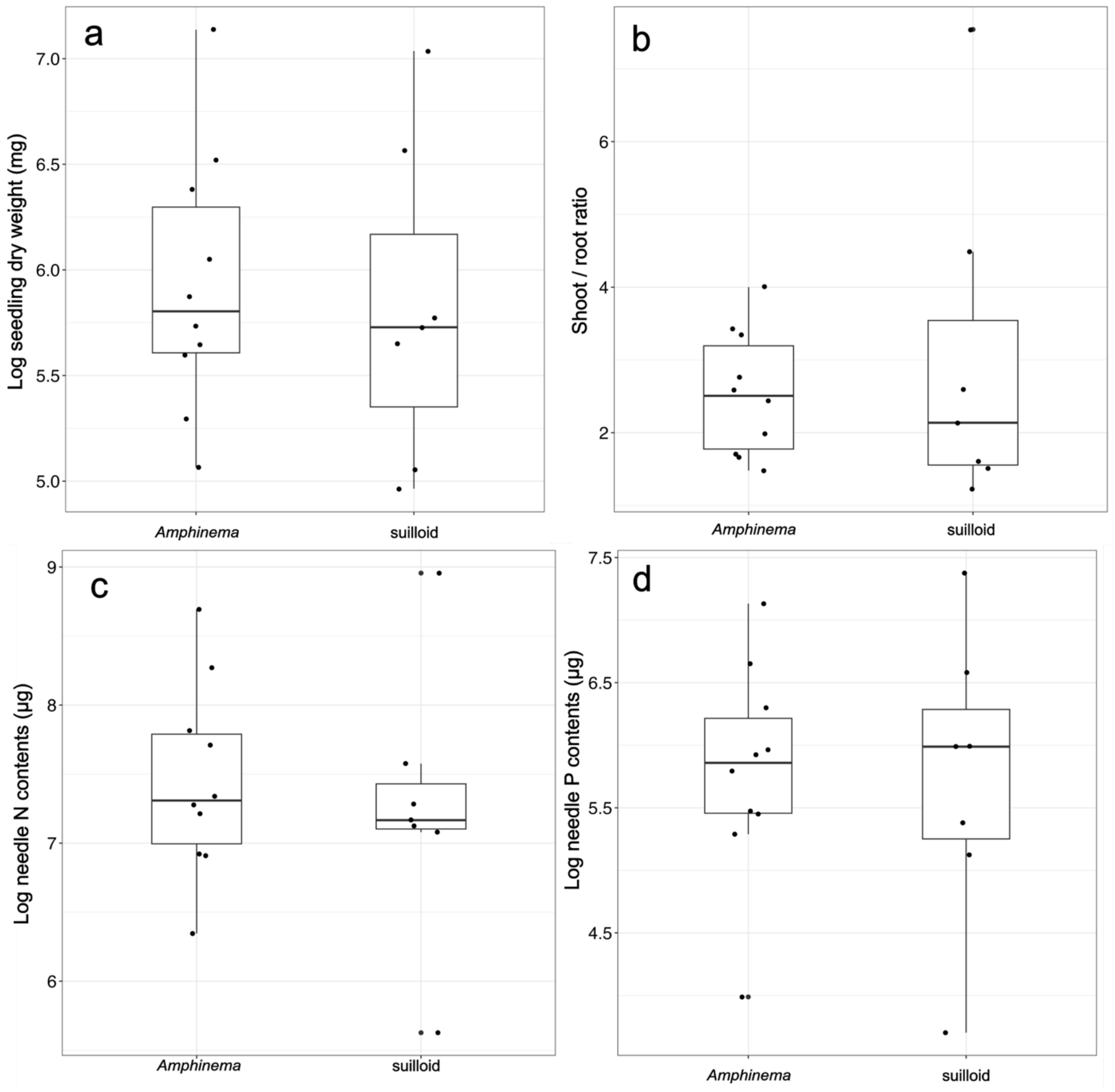

Fig. S1 Effects of ectomycorrhizal lineages on the growth and nutrient status of 1- to 2-year-old *Pinus thunbergii* seedlings in a volcanic mudflow site 6 years after the 2015 eruption on Kuchinoerabu Island. a: Log seedling dry weight. b: Shoot/root ratio. c: Log needle nitrogen contents. d: Log needle phosphorus contents. The lineage *Amphinema* and suilloid include five *Amphinema* spp. and *Rhizopogon roseolus* / *Suillus granulatus*, respectively.
